# RETRACTION: Efficacy of Home‐Based Exercise Programme on Physical Function After Hip Fracture: A Systematic Review and Meta‐Analysis of Randomised Controlled Trials

**DOI:** 10.1111/iwj.70637

**Published:** 2025-04-23

## Abstract

**RETRACTION:** ChenB. , HuN. , and TanJ.‐H. , “Efficacy of Home‐Based Exercise Programme on Physical Function After Hip Fracture: A Systematic Review and Meta‐Analysis of Randomised Controlled Trials,” International Wound Journal 17, no. 1 (2020): 45–54, 10.1111/iwj.13230.31714005 PMC7948621

The above article, published online on 12 November 2019, in Wiley Online Library (http://onlinelibrary.wiley.com/), has been retracted by agreement between the journal Editor in Chief, Professor Keith Harding; and John Wiley & Sons Ltd. Following an investigation by the publisher, all parties have concluded that this article was accepted solely on the basis of a compromised peer review process. The editors have therefore decided to retract the article. The authors did not respond to our notice regarding the retraction.

**RETRACTION:** B. Chen , N. Hu , and J.‐H. Tan , “Efficacy of Home‐Based Exercise Programme on Physical Function After Hip Fracture: A Systematic Review and Meta‐Analysis of Randomised Controlled Trials,” International Wound Journal 17, no. 1 (2020): 45–54, 10.1111/iwj.13230.31714005 PMC7948621

The above article, published online on 12 November 2019, in Wiley Online Library (http://onlinelibrary.wiley.com/), has been retracted by agreement between the journal Editor in Chief, Professor Keith Harding; and John Wiley & Sons Ltd. Following an investigation by the publisher, all parties have concluded that this article was accepted solely on the basis of a compromised peer review process. The editors have therefore decided to retract the article. The authors did not respond to our notice regarding the retraction.

